# Gastric perforation biopsy: is it obsolete?

**DOI:** 10.1007/s00423-024-03325-9

**Published:** 2024-04-27

**Authors:** Petre Francois Steyn, Otto Karusseit

**Affiliations:** Department of Surgery, University of Pretoria Medical School, Pretoria, South Africa

**Keywords:** Peptic ulcer, Perforation, Biopsy, Histology

## Abstract

**Purpose:**

The aim of the study was to test the established hypothesis that biopsies of spontaneous gastric perforations should be taken to rule out cancer.

**Methods:**

A prospective observational study was performed. Consecutive patients with spontaneous gastric perforation were included. Biopsies of the edges of the perforation were submitted for histological evaluation. The epithelial type as well as the nature of the pathology were evaluated.

**Results:**

Sixty-eight patients were included. Eight (12%) biopsies revealed duodenal origin. Sixty (88%) biopsies revealed gastric mucosa of which 33 (48%) could be specifically typed. All biopsies revealed benign ulceration. No malignancies were detected in these biopsies or on subsequent gastroscopic follow up.

**Conclusion:**

This study suggests that routine intraoperative biopsy of gastric perforation may be questioned. Biopsy is probably better performed endoscopically after recovery.

## Introduction

Perforation of a peptic ulcer is an important complication of one of the most common illnesses that beset modern man. It is currently the most common indication for surgery in patients with peptic ulcer disease (PUD)[1]. Since the introduction of effective acid suppressants and successful endoscopic haemostasis, surgery for peptic ulcers has become an exceptional necessity. In the case of perforation of a duodenal ulcer the surgery that is required is a simple closure in most cases [2]. In gastric ulcer the same usually suffices. A debate revolves around the possibility of perforation of a gastric carcinoma [3]. The incidence of cancer is reported to be from 4.2% [4] to 14% [5]. of gastric perforations. In a previous era intra-operative biopsy of a gastric perforation was required practice in order not to overlook a malignancy [6]. However, the use of endoscopy and biopsy in current practice has greatly facilitated the diagnosis of gastric cancer, whether before or after perforation. The current mainstream guidelines no longer prescribe intra-operative biopsy [7] but it is still recommended by some, [8–10. and widely performed [9–17].

Given the facility and accuracy of endoscopic diagnosis, the question is whether routine biopsy of gastric perforation is still necessary, or useful, in modern surgical practice. We undertook a study of routine biopsy of gastric perforations in order to determine the benefit of intra-operative biopsy in diagnosing the presence of cancer.

## Materials and methods

A prospective observational study was undertaken at Steve Biko Academic Hospital, which is part of the training platform of the University of Pretoria. Consecutive patients with a diagnosis of a spontaneous hollow viscus perforation were approached pre-operatively for inclusion. Those in whom gastric perforation was diagnosed at surgery were included. Biopsies were taken of the edge of the perforation including the adjacent mucosa with fine dissecting scissors or a fine-bladed scalpel. When deemed necessary the ulcer was excised. Biopsy specimens were placed in formalin and submitted for routine histological examination. All patients who proved to have gastric perforation were subjected to follow-up endoscopy.

The patients gave written informed consent for inclusion. The study was approved by the Research Ethics Committee of the Faculty of Health Sciences of the University of Pretoria. (Reference no. 235/2010/2015).

## Results

Sixty-eight patients were included in the study. Forty-eight (70%) were males and the mean age was 45 years (range 16–89 years). The operations were almost all performed by laparotomy by surgical registrars who were at varying stages of training. Most of the perforations were clearly-demarcated round defects less than 1 cm in diameter. In one case a large gastric ulcer, and in another case a stomal ulcer, were excised and reconstruction performed by consultants. Apart from these two cases all perforations were closed by simple omental patch with or without prior suturing. All patients survived.

The results of histological examination of the biopsies are shown in Table 1. Of the 68 cases deemed by the surgeon to be gastric ulcer, eight (12%) yielded duodenal histology, exhibiting Brunner glands. Thirty-three biopsies (48%) yielded gastric type mucosa while 27 (40%) did not reveal a definable mucosal type. The latter consisted of varying combinations of detached mucosa, granulation tissue, necrotic debris and inflammatory exudate, but no Brunner glands. All biopsies revealed signs of acute or chronic inflammation, with combinations of inflammatory cell infiltration, granulation tissue and fibrosis. These were all designated by the pathologist as benign peptic ulcers. No malignancies were diagnosed on these biopsies, or on follow-up gastroscopy. Two patients subsequently required gastrectomy for recalcitrant benign ulcers. After a minimal follow up of five years no patients have been diagnosed with gastric cancer.

**Table 1:** Gastric perforation biopsies. (N68)

Histology	n(%)
Gastric mucosa	60 (88)
Definable	33 (48)
Indefinable	27 (40)
Intestinal metaplasia	2 (3)
Duodenal mucosa	8 (12)
Benign peptic ulcer	68 (100)
Malignancy	0

## Discussion

We performed a series of consecutive intra-operative biopsies of gastric perforations. We aimed to determine the utility of such biopsies in diagnosing the pathology involved in the perforation. The practice of perforation biopsies dates from the pre-endoscopic era in which histological diagnoses before and/or after non-resectional surgery were necessarily unknown [18, 19]. Because gastric cancer can penetrate and perforate the gastric wall, the concern of missing a malignancy was always present [20]. This concern was incidentally addressed by the practice of performing definitive ulcer surgery for perforation [21]. This was especially recommended for gastric ulcer perforation because of poor results obtained by non-definitive procedures. This practice was also propagated by Hodnett et al., who nevertheless recommended that, in the absence of gastric resection, at least a biopsy of the perforation should be taken in order to avoid missing malignant perforation [22].

In the modern era of precise diagnosis and high cure rates for peptic ulcer disease, definitive surgery has become almost obsolete. Perforation is managed by one or other omental patch procedure in most cases. In situations where it is technically necessary resection should be performed. The World Society of Emergency Surgery (WSES) does not supply guidelines on biopsy, but also does not recommend routine gastric perforation biopsy [7]. However, the fear of missing a perforated cancer still lingers in surgical practice. Recent publications on perforations commonly recommend routine perforation biopsy [8]. Some recommend biopsy even “in a benign looking condition” [9]. The more extensive practice of “four quadrant biopsies” is also still recommended by some [12]. Some authors recommend biopsy of specific ulcer types such as gastric non-antral (Type 4) ulcer, large ulcers and those in older patients [3]. Immediate diagnosis by frozen section pathological evaluation is recommended by some when available [3, 7, 20]. Given the usual circumstances of these procedures, pathological services are seldom available.

No publications address the technical aspects of performing perforation biopsy and the consequent quality and accuracy of the material obtained. A substantial proportion of perforations deemed to be of the stomach in the current study were, in fact, of the duodenum. In these cases, the operators erroneously perceived the perforations to be proximal to the pylorus. Biopsy in these cases would be considered to be inappropriate. In addition, a significant proportion of biopsies in this study contained poor diagnostic material. Just about half of the biopsies yielded clearly-definable material as had been intended. The question of the possible harmful consequences of performing perforation biopsies is not addressed in the literature. It would seem that a tricky procedure in pathologically unstable tissue may aggravate the problem. The prudent surgeon may choose to procrastinate.

There are strong recommendations that biopsies should be obtained from all gastric ulcers to rule out malignancy [23, 24], especially in the case of a non-healing ulcer. In the case of non-perforated ulcers this is done endoscopically in otherwise symptomatic patients. This option is always available in the case of the patient with a perforated ulcer once the acute illness has been stabilized. Although it has not been tested, the quality of endoscopic biopsies in the intact stomach is likely to be superior to that of intra-operative biopsies of a perforation. The quality of perforation biopsies may be inherently problematic, as evidenced by the results of the current study which yielded a high proportion of suboptimal material. Thus, the question of the ideal time and circumstance to take biopsies of a gastric ulcer that has perforated is unanswered.

The appropriate response to an unexpected malignant PPU biopsy is uncertain. A perforated gastric carcinoma is classified as a T4 lesion. The outcome of either emergency or later elective cancer operations is generally poor [20]. The overall 30 days mortality of 13 patients in one study who underwent a local procedure or resection was 46% [4]. R0 resection in appropriate circumstances does yield better short-term results [25]. However, in an emergency situation, the nodal and metastatic status of a cancer are unknown.

The absence of any malignancies in the current study is contrary to other published series which consistently report cancer in a small proportion of cases. An explanation may be that patients with possible visceral perforation, such as those in this study, are not treated at community or any other level of hospital in our system, but only at tertiary hospitals. There is therefore no selection bias in this study, which there may be in some studies reported by specialized centres. In up to 40% of cases of gastric cancer perforation reports the diagnosis of malignancy is known before the event [19]. In addition, most reports are compiled from retrospective data, which also introduces the possibility of bias. The current study was performed prospectively on unselected patients referred to a tertiary care centre. It yielded only benign pathology. However, a limitation of this study is that the sample size is not large enough to make authoritative surgical practice recommendations. Additionally, the sample size was affected by the proportion of inappropriate duodenal histology.

## Conclusion

We performed a prospective consecutive series of biopsies of spontaneous gastric perforations. A significant proportion inadvertently yielded anatomically incorrect or poorly-diagnosable material, denoting the inefficacy of the practice. All biopsies revealed benign ulceration and no malignancies were detected. While negative biopsies are reassuring in some circumstances, the practice of performing routine intra-operative gastric perforation biopsies for cancer detection may be questioned. Initial perforation closure followed by later endoscopic biopsy seems to be the rational approach to gastric perforation in modern surgical practice.

## Data Availability

No datasets were generated or analysed during the current study.
